# Genomic Characterization of Upper-Tract Urothelial Carcinoma in Patients With Lynch Syndrome

**DOI:** 10.1200/PO.17.00143

**Published:** 2018-01-23

**Authors:** Timothy F. Donahue, Aditya Bagrodia, François Audenet, Mark T.A. Donoghue, Eugene K. Cha, John P. Sfakianos, Dahlia Sperling, Hikmat Al-Ahmadie, Mark Clendenning, Christophe Rosty, Daniel D. Buchanan, Mark Jenkins, John Hopper, Ingrid Winship, Allyson S. Templeton, Michael F. Walsh, Zsofia K. Stadler, Gopa Iyer, Barry Taylor, Jonathan Coleman, Noralane M. Lindor, David B. Solit, Bernard H. Bochner

**Affiliations:** **Timothy F. Donahue**, **François Audenet**, **Mark T.A. Donoghue**, **Eugene K. Cha**, **Dahlia Sperling**, **Hikmat Al-Ahmadie**, **Michael F. Walsh**, **Zsofia K. Stadler**, **Gopa Iyer**, **Barry Taylor**, **Jonathan Coleman**, **David B. Solit**, and **Bernard H. Bochner**, Memorial Sloan Kettering Cancer Center; **John P. Sfakianos**, Mount Sinai Hospital; **Aditya Bagrodia**, University of Texas Southwest Medical Center, Dallas, TX; **Mark Clendenning**, **Christophe Rosty**, **Daniel D. Buchanan**, **Mark Jenkins**, **John Hopper**, and **Ingrid Winship**, University of Melbourne, Parkville, Victoria, Australia; **Allyson S. Templeton**, Fred Hutchinson Cancer Research Center, Seattle, WA; and **Noralane M. Lindor**, Mayo Clinic, Scottsdale, AZ.

## Abstract

**Purpose:**

Patients with Lynch syndrome (LS) have a significantly increased risk of developing upper-tract urothelial carcinoma (UTUC). Here, we sought to identify differences in the patterns of mutational changes in LS-associated versus sporadic UTUCs.

**Patients and Methods:**

We performed targeted sequencing of 17 UTUCs from patients with documented LS-associated germline mutations (LS-UTUCs) using the Memorial Sloan Kettering Integrated Molecular Profiling of Actionable Cancer Targets targeted exon capture assay and compared the results with those from a recently characterized cohort of 82 patients with sporadic UTUC.

**Results:**

Patients with LS-UTUC were significantly younger, had had less exposure to tobacco, and more often presented with a ureteral primary site compared with patients with sporadic UTUC. The median number of mutations per tumor was significantly greater in LS-UTUC tumors than in tumors from the sporadic cohort (58; interquartile range [IQR], 47-101 *v* 6; IQR, 4-10; *P* < .001), as was the MSIsensor score (median, 25.1; IQR, 17.9-31.2 *v* 0.03; IQR, 0-0.44; *P* < .001). Differences in the genetic landscape were observed between sporadic and LS-associated tumors. Alterations in *KMT2D*, *CREBBP*, or *ARID1A* or in DNA damage response and repair genes were present at a significantly higher frequency in LS-UTUC. *CIC*, *NOTCH1*, *NOTCH3*, *RB1*, and *CDKN1B* alterations were almost exclusive to LS-UTUC. Although *FGFR3* mutations were identified in both cohorts, the R248C hotspot mutation was highly enriched in LS-UTUC.

**Conclusion:**

LS- and sporadic UTUCs have overlapping but distinct genetic signatures. LS-UTUC is associated with hypermutation and a significantly higher prevalence of *FGFR3* R248C mutation. Prospective molecular characterization of patients to identify those with LS-UTUC may help guide treatment.

## INTRODUCTION

Lynch syndrome (LS) is an autosomal dominant cancer predisposition syndrome caused by germline mutations in the mismatch repair (MMR) genes *MLH1*, *MSH2*, *MSH6*, or *PMS2.* Patients with LS have an increased risk of developing a variety of tumors, particularly those arising in the colon, but also extracolonic cancers, including urothelial carcinomas (UCs).^1-3^ UC is the third most frequent malignancy in LS, occurring in approximately 5% of patients.^3,4^ Patients with LS have up to a 22-fold greater risk of developing upper-tract urothelial carcinoma (LS-UTUC) over the general population and a median age of onset 10 to 15 years earlier than patients with sporadic UTUC.^2-5^

Although both UC of the bladder (UCB) and UTUC are believed to arise from a common precursor cell population within the urothelium, increasing evidence suggests that these two malignancies represent different disease entities from both clinicopathologic and genetic perspectives.^6-10^ In support of this concept, tumor genomic sequencing of UCB and UTUC has identified distinct mutational profiles between these two urothelial malignancies.^11,12^

Here, we sought to determine whether patients with UTUC with known germline mutations uniformly harbored loss of heterozygosity of the wild-type allele of the gene that was the basis of their LS diagnosis and whether their tumors exhibited a pattern of hypermutation consistent with MMR deficiency. We also sought to identify differences in the patterns of mutational changes in LS-UTUC versus sporadic UTUC that may represent potential therapeutic targets.

## PATIENTS AND METHODS

### Study Samples

Upper-tract tumors (n = 21) and normal samples were collected from patients with a known germline mutation in an LS-associated gene through a collaborative effort between the Colon Cancer Family Registry (CCFR) and Memorial Sloan Kettering Cancer Center. The CCFR activities are conducted across six multiple–principal investigator sites and six subaward sites: University of Melbourne (Melbourne, Victoria, Australia), Fred Hutchinson Cancer Research Center (Seattle, WA), Sinai Health System (comprising Mount Sinai Hospital and Lunenfeld-Tanenbaum Research Institute [Toronto, Ontario, Canada]), Mayo Clinic (Mayo Clinic Arizona [Scottsdale, AZ] with a subaward to Mayo Clinic Minnesota [Rochester, MN]), Cedars-Sinai Consortium (comprising Cedars-Sinai Medical Center [Los Angeles, CA] with subawards to Cleveland Clinic [Cleveland, OH], University of Minnesota [Minneapolis, MN], Dartmouth College [Hanover, NH], and University of Virginia [Charlottesville, VA]), and University of Hawaii (University of Hawaii Medical Center [Honolulu, HI] with a subaward to Cancer Prevention Institute of California [Fremont, CA]). This was formed as a resource to support studies on the etiology, prevention, and clinical management of colorectal cancer. The resource comprises data and biospecimens from approximately 40,000 participants from 14,000 families recruited from 1998 to 2016.^13^ For this study, the inclusion criteria were proven pathogenic germline mutation in one of the DNA MMR genes *MLH1*, *MSH2*, *MSH6*, or *PMS2*; diagnosis of UTUC confirmed by expert pathologic examination; and availability of archival tissue blocks for genetic analysis. Study approval was obtained from the institutional review board or human research ethics committee of each participating institution. A previously characterized cohort of 82 patients with presumed sporadic UTUC treated with radical nephroureterectomy was used as a comparison group.^11^

### Sample Preparation

Germline DNA was extracted from peripheral blood lymphocytes provided by the CCFR for each patient. Paraffin-embedded tumor blocks were obtained. Hematoxylin and eosin–stained sections were prepared from each block and reviewed by a board-certified pathologist to confirm the histologic diagnosis and tumor grade. For four tumors obtained from the Mount Sinai Hospital, previously extracted tumor genomic DNA was provided. For these cases, pathologic review was performed on high-resolution digital images of the hematoxylin and eosin–stained sections. No patient had a dominant variant histologic subtype. When not provided by the CCFR, DNA was extracted from paraffin sections as previously described.^12^ Clinical and demographic information were obtained from the prospectively maintained registry of the CCFR.

### Targeted Sequencing

All protein-coding exons of 341 cancer-associated genes were sequenced using the Memorial Sloan Kettering Integrated Molecular Profiling of Actionable Cancer Targets (MSK-IMPACT) assay as previously described (Data Supplement).^14^ All candidate mutations and indels were reviewed manually. The accumulated sequence coverage for each exon was compared in tumor and matched germline samples. Coverage ratios ≥ 3× were defined as amplifications, and ratios ≤ 0.3× were defined as deletions, whereas coverage ratios ≥ 2× and ≤ 0.5× were defined as gains and losses, respectively.

To determine allelic configurations, total and allele-specific copy-number states were inferred for all tumor samples using FACETS (version 0.5.6).^15^ The proportions of unstable microsatellites were quantitated using MSIsensor (version 0.2).^16^ Microsatellite instability (MSI) score was defined as the percentage of unstable microsatellite sites divided by total number of microsatellite sites surveyed. Signature decomposition analysis^17,18^ was performed for all tumor samples with ≥ 10 single-nucleotide somatic mutations. From the somatic mutations in an individual tumor sample, contributions were inferred from known mutational signatures, which are probability distributions over the nucleotide change and flanking 5′ and 3′ nucleotide context of each mutation. The quasi *P* value for a particular signature was calculated as the fraction of samples with a signature proportion greater than a precalculated noise threshold (1 × 10^−5^). Signatures representing ≥ 15% mutations and with a significant quasi *P* value (< .05) were plotted.

### Statistical Analysis

Pearson’s χ^2^ test or Fisher’s exact test were used to test statistical significance of differences between clinical and demographic variables. Bivariable comparisons of individual mutation frequencies by cohort were performed using Fisher’s exact test. Counts of gains, losses, and total copy-number alterations were analyzed using negative binomial regression. *P* values < .05 were considered statistically significant. All analyses were conducted using R software (version 2.13.1; R Foundation, Vienna, Austria).

## RESULTS

### Genomic Characterization of LS-UTUC

**Fig 1.**
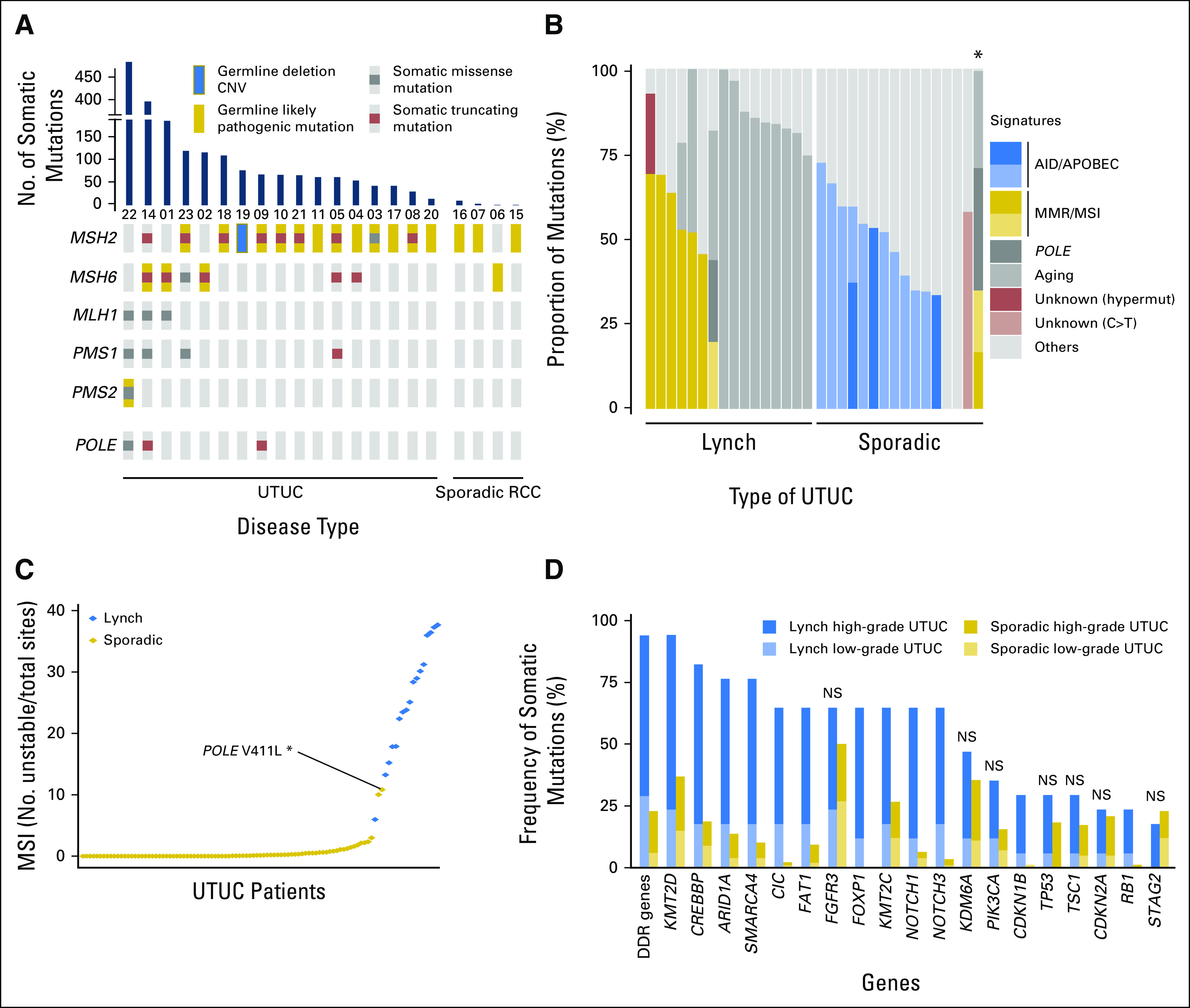
(A) Oncoprint of germline and somatic mutations in mismatch repair (MMR) genes and in *POLE* in the Lynch cohort. (B) Mutational decomposition analysis of the samples with ≥ 10 somatic mutations. (*) Hypermutated sporadic upper-tract urothelial carcinoma (UTUC) tumor with a hotspot mutation in *POLE*. (C) Microsatellite instability (MSI) sensor scores across the two cohorts. (D) Frequency of somatic alterations in Lynch syndrome–associated (n = 17) and sporadic UTUC (n = 82) cohorts. AID/APOBEC, activation-induced cytidine deaminase/apolipoprotein B mRNA-editing enzyme catalytic polypeptide; CNV, copy-number variation; DDR, DNA damage repair; NS, non significant; RCC, renal cell carcinoma.

**Fig A1.**
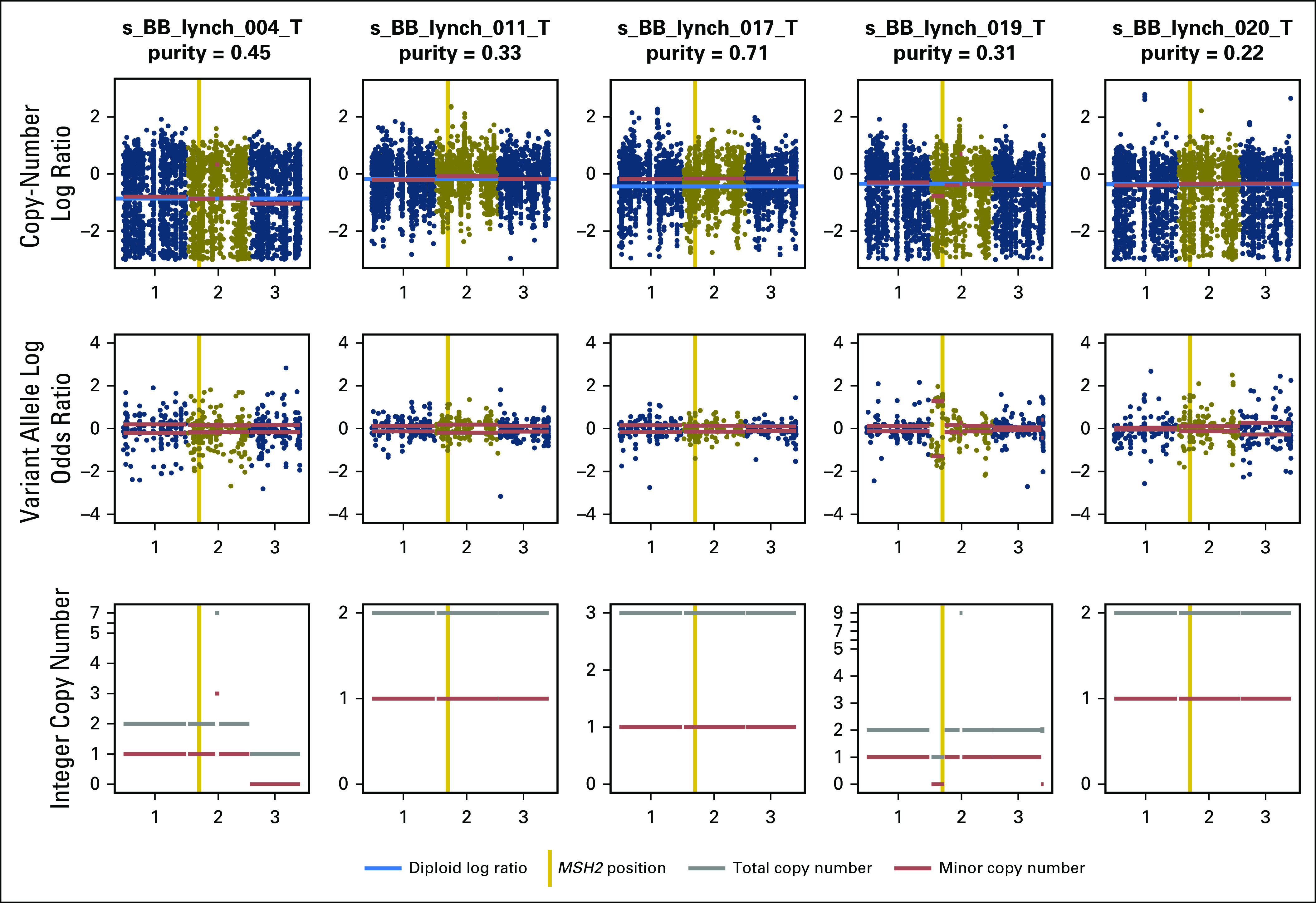
FACETS analysis of five samples without somatic alterations corresponding to germline mutations identified in mismatch repair genes. Sample s_BB_lynch_019_T was found to present loss of heterozygosity in *MSH2*.

Of the 21 tumors analyzed, four were reclassified as renal cell carcinoma (RCC) on central pathologic review. Genomic DNA from all 21 sample tumors (LS-UTUC, n = 17; RCC, n = 4) and matched normal peripheral blood lymphocytes were analyzed for alterations in 341 cancer-associated genes. The average sequencing coverage was 315× for all targeted exons across all 21 patients, with average coverage of 251× per tumor (Data Supplement). In all 21 patient cases, we confirmed the presence of a deleterious germline alteration in an LS-associated gene. In patients with LS-UTUC, *MSH2* was the most common germline alteration (13 [76%] of 17), followed by *MSH6* (three [18%] of 17) and *PMS2* (one [6%] of 17). No germline alterations were identified in *MLH1* or *PMS1*. Thirteen (76%) of 17 patients had loss of heterozygosity in the DNA repair genes that corresponded to the germline alterations identified in their germline DNA (Fig 1A; Appendix Fig A1).

A total of 1,834 somatic coding alterations were identified across all patients with LS-UTUC, involving 300 different genes (Data Supplement). The median number of somatically altered genes per tumor was 47 (range, nine to 204). Characteristic of MMR-deficient neoplasms, the mean MSIsensor score was high (median, 25.1; range, 6 to 37.7), with an excess of frameshift mutations (142 [8%] in 1,834), whereas copy-number alterations were uncommon.^19^ Using mutational decomposition analysis in samples with ≥ 10 somatic mutations, we identified evidence of MSI/MMR signatures in seven tumors, whereas the mitotic clock/aging signature was predominant in nine (Fig 1B). Notably, the four RCCs found to arise in the patients with LS had low mutation rates (median, two; range, one to nine), low MSIsensor scores (median, 4.7; range, 3 to 11.6), and no evidence of loss of heterozygosity of their LS-associated germline mutation, suggesting that the pathogenesis of these tumors was biologically independent of their LS diagnosis.

**Fig A2.**
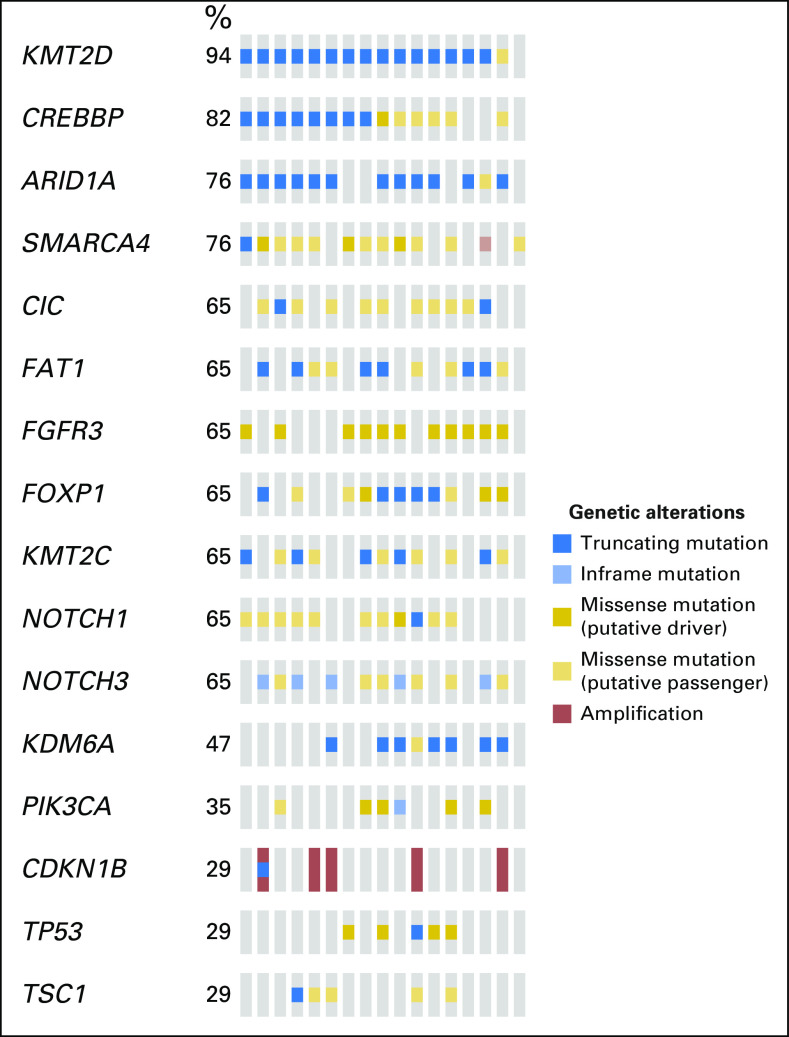
Oncoprint of the most frequent genes altered in the Lynch cohort.

The most frequently mutated genes in the 17 LS-UTUC tumors (present in > 10 patients) in decreasing frequency were *KMT2D*, *CREBBP*, *ARID1A*, *SMARCA4*, *CIC*, *FAT1*, *FGFR3*, *FOXP1*, *KMT2C*, *NOTCH1*, and *NOTCH3* (Appendix Fig A2). With the exception of *CIC*, *FOXP1*, and *KMT2C*, these genes were also those with the highest mutation rate (> 0.002 mutations per nucleotide; Data Supplement). *TP53* was mutated in a smaller subset of carcinomas (five [29%] of 17), and in four of these, the *TP53* mutation co-occurred with *FGFR3* mutations. *RB1* alteration was found in four (24%) of 17. Of interest, although only 11 focal amplifications were identified within the 17 LS-UTUCs, five of these 11 amplification events involved *CDKN1B.* Moreover, *CDKN1B* amplification showed a tendency toward mutual exclusivity with both *FGFR3* and *TP53* mutations.

### Comparison of Genomic Profiles of LS-UTUC and Sporadic UTUC

To identify potential differences in the mutational landscape between LS-UTUC and sporadic UTUC, we compared the results of the LS-UTUC cohort with those from a previously reported analysis by our group of 82 patients with sporadic UTUC. One patient in this prior cohort had an ultramutated phenotype (422 mutations and no focal copy-number alterations) attributable to a hotspot mutation in *POLE*.^11^ Patient demographic and clinicopathologic characteristics of the two groups are listed in Table 1. Compared with those with sporadic UTUC, patients with LS were significantly younger (median age, 61 years; interquartile range [IQR], 53–66 years *v* 68.5 years; IQR, 63-75 years; *P* = .005), had had less exposure to tobacco (47% *v* 73%; *P* = .035), and had a different distribution of primary tumor location (47% ureteral tumor in LS *v* 18% in the sporadic cohort; *P* = .011).

**Table 1.**
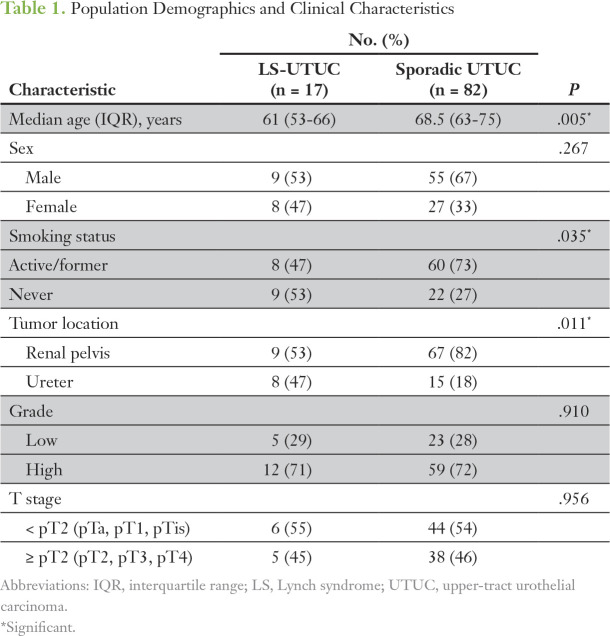
Population Demographics and Clinical Characteristics

As expected, the median number of mutations per sample for LS-UTUC was significantly greater than that seen in the sporadic cohort (58; IQR, 47-101 *v* six; IQR, 4–10; *P* < .001), and the MSIsensor score was also significantly higher (median, 25.1; IQR, 17.9-31.2 *v* 0.03; IQR, 0-0.44; *P* < .001; Fig 1C). Most of the sporadic samples with ≥ 10 somatic mutations had an AID/APOBEC (activation-induced cytidine deaminase/apolipoprotein B mRNA-editing enzyme catalytic polypeptide) signature (12 of 16; Fig 1B).

We next compared the frequencies of genetic alterations in the most commonly altered genes (Table 2; Fig 1D) between the two tumor cohorts. This revealed that, although the mutational landscapes overlapped, some genes were targets of somatic alteration in both cohorts; however, the frequency of alteration was significantly higher in LS-UTUC (ie, *KMT2D*, *CREBBP*, *ARID1A*, and *SMARCA4*). Moreover, some genes were somatic targets almost exclusively in the LS cohort. Examples of this include *CIC*, *FOXP1*, *NOTCH1*, *NOTCH3*, or *RB1*, each of which harbored somatic alterations in nonoverlapping subsets in < 6% of sporadic UTUCs versus 24% to 65% in LS-UTUCs (*P* < .001). Copy-number alterations such as *CDKN1B* amplification were also unique to LS-UTUCs (five of 17 *v* zero of 82; *P* < .001). Of note, alteration in at least one of the DNA damage response and repair genes (*ERCC2*, *ERCC3*, *ERCC4*, *ERCC5*, *BRCA1*, *BRCA2*, *RAD50*, *RAD51*, *RAD51B*, *RAD51C*, *RAD51D*, *RAD52*, *RAD54L*, *NBN*, *MRE11A*, *ATM*, *ATR*, *MDC1*, *CHEK1*, *CHEK2*, *PALB2*, *BRIP1*, *FANCA*, *FANCC*, *BLM*, *MUTYH*, *RECQL4*, *PARP1*, and *POLE*) was found in 94% of the patients with LS, compared with 23% in the sporadic cohort (*P* < .001). When adjusting for grade, most of the differences between the two cohorts remained significant, although the frequency of gene alterations was different between low- and high-grade tumors (Table 2).

**Table 2.**
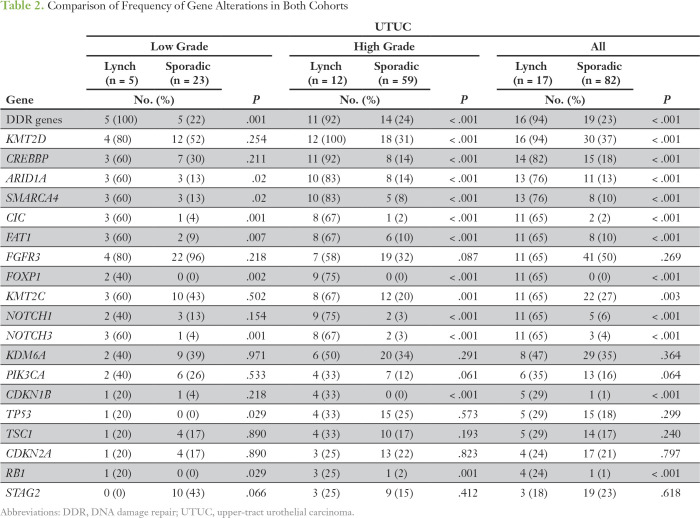
Comparison of Frequency of Gene Alterations in Both Cohorts

**Fig 2.**
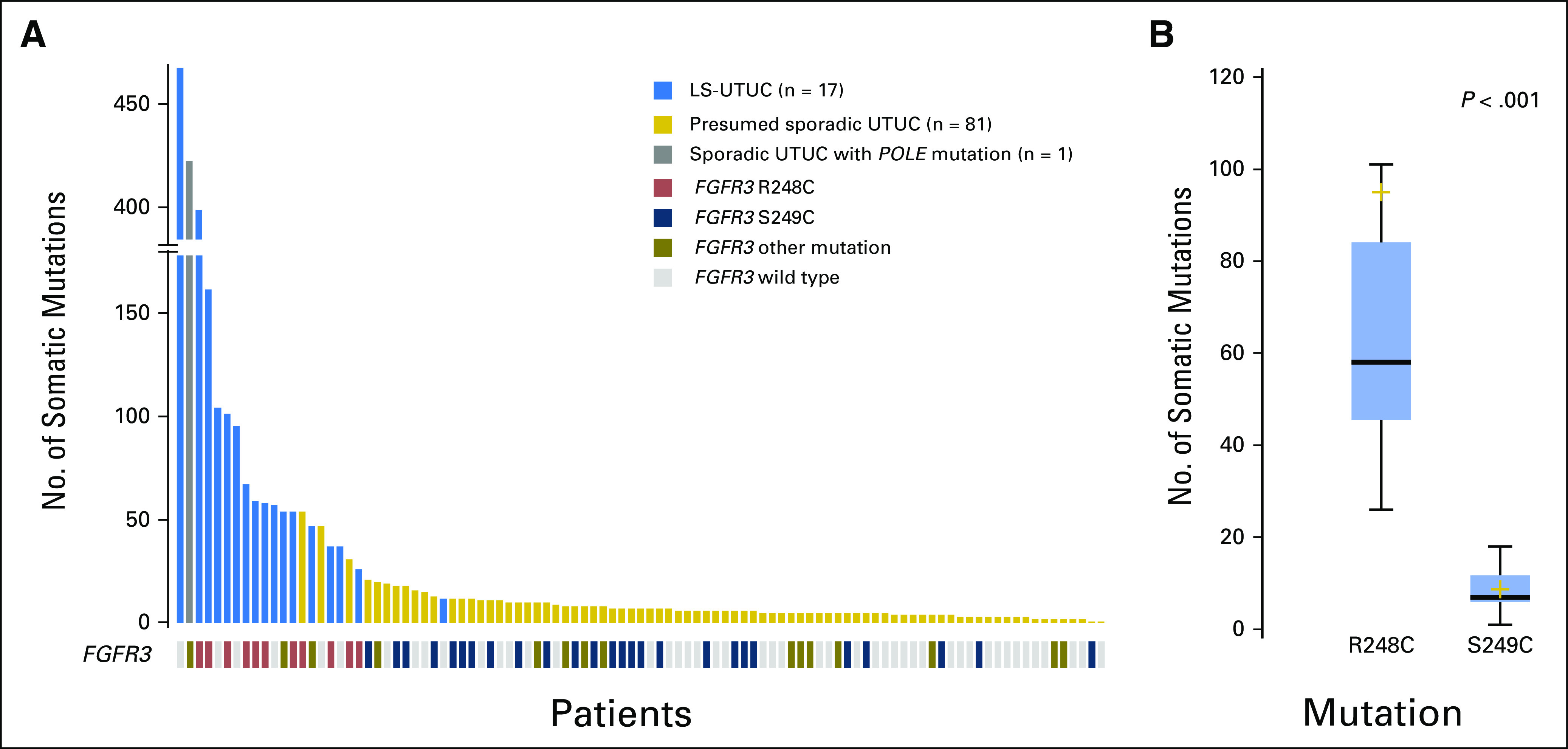
(A) Number of somatic alterations in Lynch syndrome–associated upper-tract urothelial carcinoma (LS-UTUC) and sporadic UTUC tumors and their association with *FGFR3* mutation status. (B) Total mutation count in patients with somatic *FGFR3* R248C and *FGFR3* S249C mutations.

**Fig A3.**
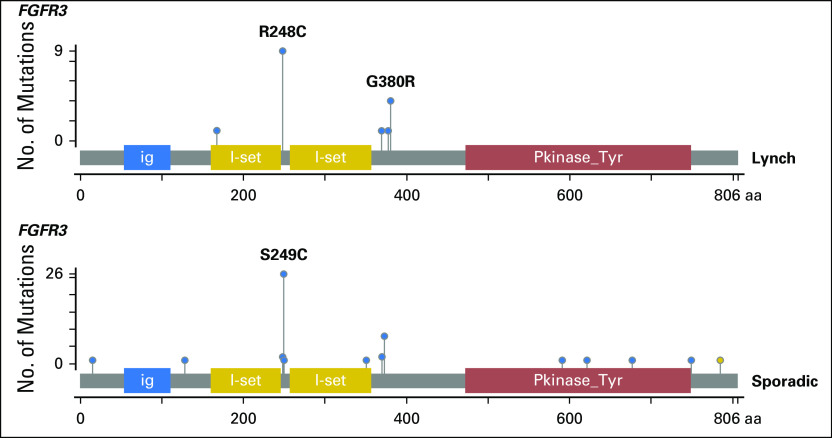
Lollipop plots of *FGFR3* mutations in the sporadic and Lynch cohorts.

Finally, we noted that both cohorts had similar frequencies of *FGFR3* mutations (LS-UTUC, 11 [65%] of 17 *v* sporadic UTUC, 41 [50%] of 82; *P* = .269; Appendix Fig A3). Not surprisingly, *FGFR3* mutations were more frequent in low-grade tumors (80% of low-grade LS-UTUCs *v* 94% of low-grade sporadic UTUCs; *P* = .218). However, whereas the most prevalent *FGFR3* hotspot mutation in sporadic UTUC was S249C (26 [63%] of 41 mutations; 13 of 22 and 13 of 19 for low- and high-grade tumors, respectively), in LS-UTUC, the *FGFR3* mutations noted were predominantly R248C (nine [82%] of 11 mutations; four of four and five of seven for low- and high-grade tumors, respectively) and to a lesser extent G380R (four [36%] of 11 mutations; two of four and two of seven for low- and high-grade tumors, respectively; Fig 2A).

Given this unexpected finding, we rereviewed all 41 *FGFR3* mutations in our sporadic cohort of 82 UTUCs and identified two carcinomas with an *FGFR3* R248C mutation. These UTUCs had 31 and 54 total somatic mutations, respectively, by MSK-IMPACT, suggesting the possibility of an MMR defect. However, their MSIsensor scores were low (0 and 0.11), and the mutational decomposition analysis revealed an AID/APOBEC signature.

### Validation of *FGFR3* R248C as Marker of LS-UTUC

**Fig 3.**
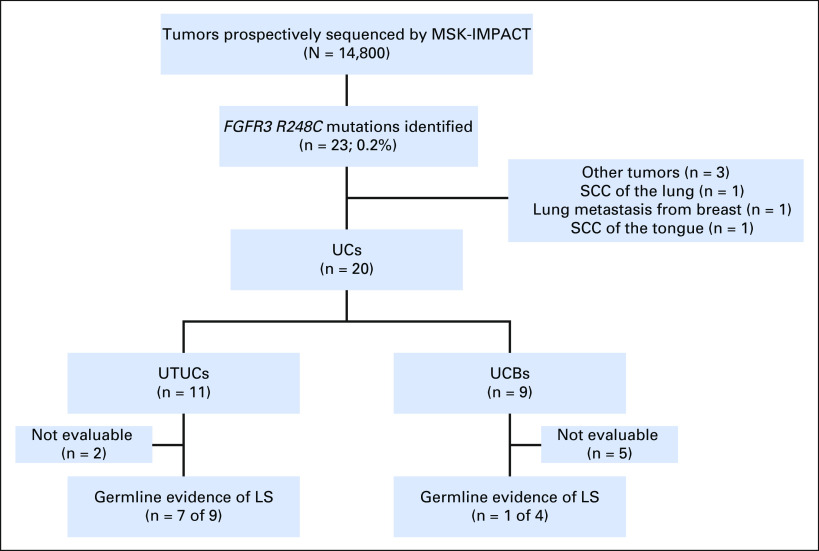
Flow chart of identification of the patients with *FGFR3* R248C mutation in the clinical Memorial Sloan Kettering Integrated Molecular Profiling of Actionable Cancer Targets (MSK-IMPACT) cohort. LS, Lynch syndrome; SCC, squamous cell carconima; UC, urothelial carcinoma; UCB, UC of the bladder; UTUC, upper tract UC.

To determine the extent to which the presence of an *FGFR3* R248C mutation may be associated with LS or an LS-like hypermutator phenotype, we queried 14,800 tumors prospectively sequenced as part of clinical care at Memorial Sloan Kettering Cancer Center using the MSK-IMPACT assay. Twenty-three patients had an *FGFR3* R248C mutation, corresponding to 11 UTUCs, nine UCBs, and three tumors with other primary sites (one squamous cell carcinoma of the lung, one lung metastasis from breast carcinoma, and one squamous cell carcinoma of the tongue; Fig 3). The 11 patients with UTUC had a median number of mutations of 41 (range, one to 414 mutations). Consent for germline genomic analysis was lacking for two patients. Of the nine evaluable patients with presumed sporadic UTUC and an *FGFR3* R248C hotspot mutation, seven displayed germline evidence of LS (six germline mutations in *MSH2* and one in *MSH6*). One of the patients without evidence of a germline mutation had a hypermutator tumor (41 mutations) with absence of MSH2 and MSH6 expression by immunohistochemistry consistent with a somatic MSI-high, LS-like phenotype.

In our study, the presence of an *FGFR3* R248C hotspot mutation was associated with a higher median number of somatic mutations compared with tumors with an *FGFR3* S249C mutation (58; IQR, 45.5-84 *v* seven; IQR, 6-11.8; *P* < .001; Fig 2B). Similarly, of the 14,800 tumors prospectively sequenced at our institution, the 23 samples with *FGFR3* R248C mutations, including 20 UCs and three other tumors, had a significantly higher median number of somatic mutations compared with that in the 84 patients with *FGFR3* S249C mutations across all tumor types (22; IQR, 8-53 *v* nine; IQR, 6-11.8; *P* < .006).

## DISCUSSION

LS is an autosomal dominant cancer predisposition syndrome attributable to germline mutations in an MMR gene. Upwards of 80% of patients with LS will develop a malignancy, with colorectal cancer being the most common.^20^ UC is the third most common site of extracolonic malignancy in the LS spectrum and occurs in approximately 5% of patients. Few studies to date have sought to define the somatic mutational profile of MMR-deficient urothelial tumors, in part because of the perception that the high mutational load would limit the ability to identify true driver events.^19^ In this study, we performed targeted sequencing of UTUC from 17 patients with known germline mutations in an LS-associated gene to characterize the genomic landscape of these tumors and compared the results with those from a cohort of 82 patients with clinically presumed sporadic UTUC.

Next-generation sequencing (NGS) can be used to discover novel, targetable, or pathogenic somatic alterations and identify patients with mutations for which therapies already exist. Although many of the driver genes identified in the LS and sporadic UTUC cohorts tended to be similar (*FGFR3*, *KDM6A*, *PIC3CA*, and *TP53*), differences in the genomic profiles were observed. As would be expected from an MSI-associated malignancy, the median number of mutations per tumor was significantly higher in LS-UTUC than sporadic UTUC (58 *v* six; *P* < .001). There was an excess of frameshift mutations (142 [8%] of 1,834), and copy-number alterations were relatively uncommon. Mutation of some genes was almost exclusive to LS-UTUC (*CIC*, *NOTCH1*, *NOTCH3*, and *RB1*). Of note, however, hypermutation or loss of heterozygosity of the LS germline mutation was not noted in the four RCC samples collected from patients with LS, suggesting that the pathogenesis of these tumors was unrelated to the LS diagnosis.

Although we were able to identify somatic alterations in most patients driving loss of heterozygosity in the germline LS genes relevant to each corresponding patient, four patients with LS had germline mutations without evidence of loss of heterozygosity as detectable by tumor DNA sequencing. All four, however, had genomic signatures consistent with MSI, suggesting that these patients may have had transcriptional silencing by promoter hypermethylation, as previously reported.^21^ Regarding the mutational decomposition analysis, only seven LS-UTUC tumors had an MMR/MSI signature. This mutational decomposition analysis only uses single-nucleotide polymorphisms, and these are not the most prevalent type of mutation present in MSI/MMR cases.^19^ This may partially explain why the decomposition analysis did not identify a strong MSI/MMR signature in some patient cases, including many with high MSIsensor scores.

Among the most striking findings in our study was the identification of an R248C hotspot mutation in *FGFR3* that may serve as a potential biomarker for LS in patients with UTUC. *FGFR3* is one of four members of the fibroblast growth factor receptor family of receptor tyrosine kinases that promote cell growth and proliferation. Activating mutations of *FGFR3* are particularly common in low-grade UCs.^22,23^ *FGFR3* R248C leads to increased FGFR3 dimer stability and constitutive receptor activation in the absence of ligand, resulting in activation of the phosphatidylinositol 3-kinase–AKT and mitogen-activated protein kinase signaling pathways.^24,25^ This mutation has already been described in UCB,^26^ but its presence in UTUC was highly associated with a significantly increased median number of somatic mutations.

Some hereditary cancers are misclassified as sporadic, although their identification might have consequences for both patients and their family members, because this information would prompt germline testing and, if positive, screening for LS-related malignancies.^27^ Our data suggest that the finding of an *FGFR3* R248C somatic mutation is a potential biomarker for LS and its identification in a patient should prompt germline testing for LS. Furthermore, recent data suggest that tumors with MMR deficiency are more responsive to immune checkpoint blockade than MMR-proficient tumors.^28^ It is possible that patients with LS-UTUC may derive greater benefit from anti–PD-1 therapy, suggesting an immediate clinical implication for screening and potential personalization of therapy in these patients.

One potential limitation of our study was the use of a targeted sequencing approach focusing on a panel of 341 cancer-related genes. A broader sequencing approach, such as whole-exome sequencing, may have identified additional genes differentially altered in sporadic and LS-UTUCs. Recently, the analysis of 28 samples of sporadic UTUC identified four RNA expression profiles with different clinical characteristics, and it would be biologically important to study how LS-UTUCs cluster within this classification.^29^ Additionally, although ours is the largest series to date to our knowledge profiling LS-UTUC using NGS, the sample size remains small, and a larger study may have revealed other significant patterns of comutated genes. Consequently, there is a need for a larger cohort with external validation.

In conclusion, we performed targeted NGS of 17 UTUCs that arose in patients with LS to compare the genetic characteristics of such tumors with those of sporadic UTUC tumors. Although the spectrums of mutated genes showed some similarities, LS-UTUCs were characterized by a unique genetic signature, consisting of hypermutation and frequent hotspot mutations at *FGFR3* R248C. The results of this study may help to differentiate between presumed sporadic UTUC and UTUC with an underlying MSI-associated germline defect, which could be used to guide specific screening and treatment recommendations.
